# Choroidal thickness estimation from colour fundus photographs by adaptive binarisation and deep learning, according to central serous chorioretinopathy status

**DOI:** 10.1038/s41598-020-62347-7

**Published:** 2020-03-27

**Authors:** Yuki Komuku, Atsuya Ide, Hisashi Fukuyama, Hiroki Masumoto, Hitoshi Tabuchi, Takeshi Okadome, Fumi Gomi

**Affiliations:** 1 Department of Ophthalmology, Hyogo College of Medicine, Nishinomiya, Japan; 2 Kwansei Gakuin University, Sanda, Japan; 3 Department of Ophthalmology, Tsukazaki Hospital, Himeji, Japan

**Keywords:** Diseases, Health care, Medical research

## Abstract

This study was performed to estimate choroidal thickness by fundus photography, based on image processing and deep learning. Colour fundus photography and central choroidal thickness examinations were performed in 200 normal eyes and 200 eyes with central serous chorioretinopathy (CSC). Choroidal thickness under the fovea was measured using optical coherence tomography images. The adaptive binarisation method was used to delineate choroidal vessels within colour fundus photographs. Correlation coefficients were calculated between the choroidal vascular density (defined as the choroidal vasculature appearance index of the binarisation image) and choroidal thickness. The correlations between choroidal vasculature appearance index and choroidal thickness were −0.60 for normal eyes (p < 0.01) and −0.46 for eyes with CSC (p < 0.01). A deep convolutional neural network model was independently created and trained with augmented training data by K-Fold Cross Validation (K = 5). The correlation coefficients between the value predicted from the colour image and the true choroidal thickness were 0.68 for normal eyes (p < 0.01) and 0.48 for eyes with CSC (p < 0.01). Thus, choroidal thickness could be estimated from colour fundus photographs in both normal eyes and eyes with CSC, using imaging analysis and deep learning.

## Introduction

Choroidal thickness is associated with the pathologies of various fundus diseases, including central serous chorioretinopathy (CSC), polypoidal choroidal vasculopathy, and myopic degeneration^1–3^. To measure the choroidal thickness of an eye, optical coherence tomography (OCT) with enhanced depth imaging^4,5^ or the swept-source system is essential^6,7^. Using these methods, many studies have found physiological and pathological changes in the choroid^8–10^.

The choroid is known to become thinner with age and increasing myopia^9,11^; such changes lead to a tigroid fundus appearance (i.e., choroidal vessels are increasingly apparent). Conversely, when the choroid becomes thickened, as in Vogt–Koyanagi–Harada disease, choroidal vessels exhibit a blurry fundus appearance^12^. Therefore, we hypothesized that fundus images could support the estimation of choroidal thickness, thereby enabling determination of which eyes might exhibit thick or thin choroid. The aim of this study was to determine whether choroidal thickness could be estimated in fundus images using image processing and deep learning methods.

## Results

### Characteristics of normal eyes and eyes with CSC in this study

We used fundus photographs and OCT images of 200 normal eyes (mean patient age 60.4 ± 15.2 years; range, 10–89 years) and 200 eyes with CSC (mean patient age 57.2 ± 11.3 years, range, 29–85). The average spherical equivalent refractive error was −1.19 ± 2.95 dioptres (D) for normal eyes and −0.17 ± 2.28 D for eyes with CSC. The average choroidal thicknesses were 267 ± 100 μm for normal eyes and 343 ± 96.4 μm for eyes with CSC (Table 1). Eyes with CSC were characterised by a significantly greater spherical equivalent refractive error and greater choroidal thickness, compared with normal eyes. There was no significant difference in patient age between the normal eyes and eyes with CSC.

**Table 1:** CSC, central serous chorioretinopathy; SERE, spherical equivalent refractive error; CT, choroidal thickness.

	Normal (n = 200)	CSC (n = 200)	P-value
age (yrs)	60.4 ± 15.2	57.2 ± 11.3	0.26
SERE (D)	−1.19 ± 2.95	−0.17 ± 2.28	0.0001
CT (μm)	267 ± 100	343 ± 96.4	<0.0001

### Binarisation image processing

**Figure 1:**
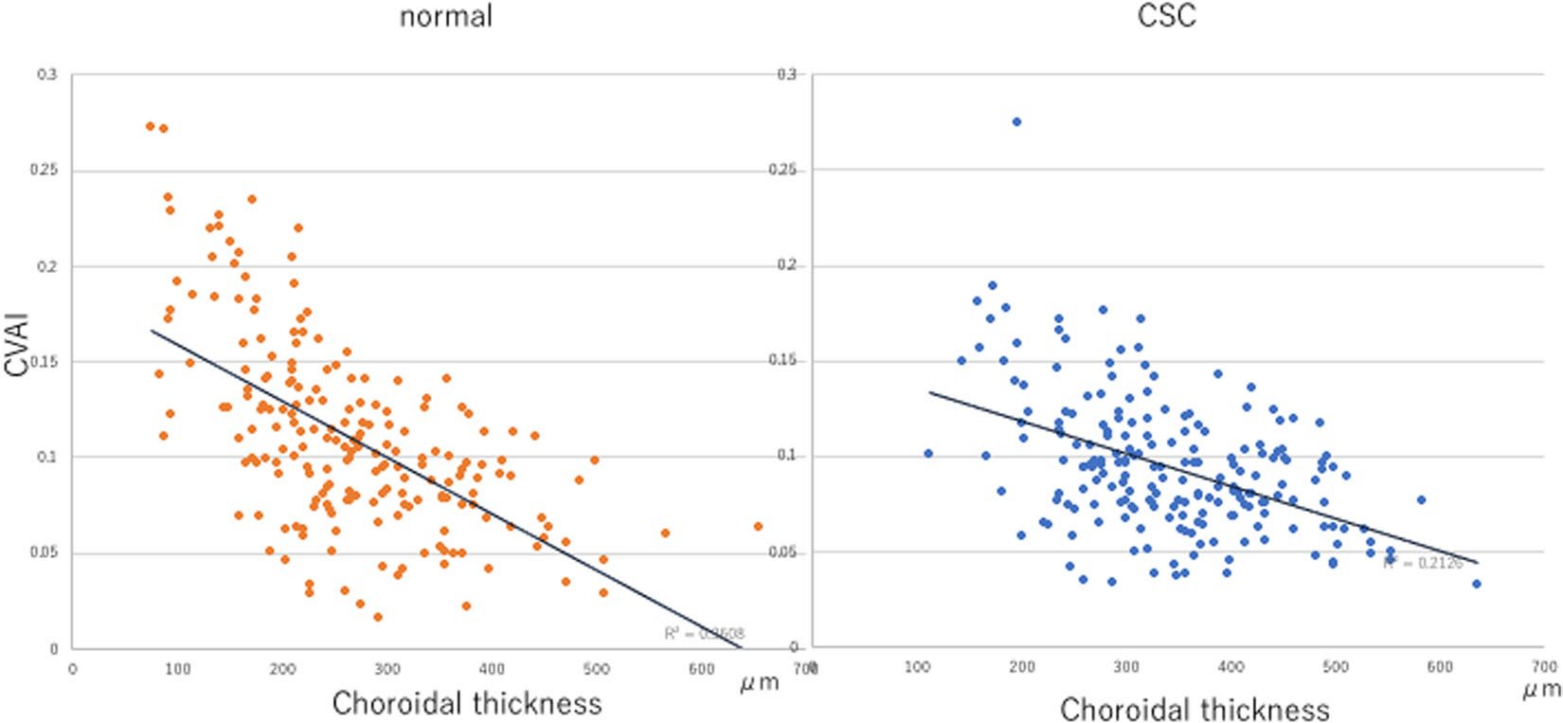
Correlation between choroidal vasculature appearance index (CVAI) and choroidal thickness in normal eyes (left) and in eyes with central serous chorioretinopathy (CSC) (right).

The average choroidal vasculature appearance index (CVAI) for normal eyes was 0.11 ± 0.05%, whereas it was 0.09 ± 0.04% for eyes with CSC; the CVAI was significantly greater in normal eyes than in eyes with CSC (p = 0.002). For normal eyes, the correlation coefficient between CVAI and choroidal thickness was −0.60 (p < 0.001); the correlation coefficients between CVAI and age, and between CVAI and spherical equivalent refractive error, were −0.04 and −0.41, respectively. For eyes with CSC, the correlation between CVAI and choroidal thickness was −0.46 (p < 0.001), the correlation between CVAI and age was −0.19, and the correlation between CVAI and spherical equivalent refractive error was −0.16 (Fig. 1).

**Figure 2:**
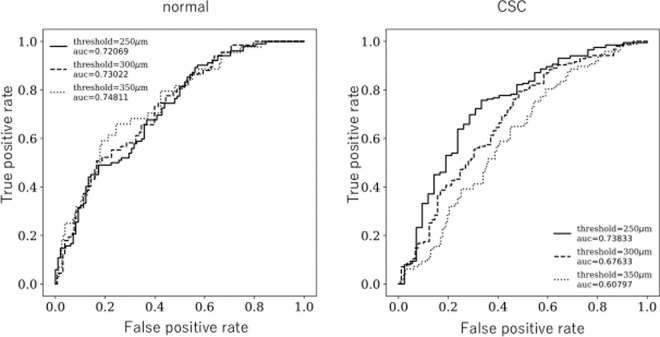
Receiver operating characteristic curve and area under the curve (AUC) obtained using choroidal vasculature appearance index (CVAI) and choroidal thickness from normal eyes (left) and eyes with central serous chorioretinopathy (CSC) (right). The formula for calculation of ‘True positive rate’ was TP/(TP + FN); the formula for calculation of ‘False positive rate’ was FP/(FP + TN). TP: True Positive Rate FN: False Negative Rate. FP: False Positive Rate TN: True Negative Rate. In normal eyes, the AUCs were 0.72, 0.73, and 0.75 with threshold values of 250 µm, 300 µm, and 350 µm, respectively. In eyes with CSC, the AUCs were 0.74, 0.68, and 0.61 with thresholds of 250 µm, 300 µm, and 350 µm, respectively.

To confirm the accuracy of the estimates of choroidal thickness, the area under the receiver operating characteristic curve (AUC) was confirmed using three threshold values: 250 μm, 300 μm, and 350 μm. The formula for calculation of ‘true positive rate’ was true positive/(true positive + false negative); the formula for calculation of ‘false positive rate’ was false positive/(false positive + true negative). For normal eyes, the AUCs were 0.72, 0.73, and 0.75 for the threshold values of 250 μm, 300 μm, and 350 μm, respectively; for eyes with CSC, the corresponding AUCs were 0.74, 0.68, and 0.61 respectively. For normal eyes, the AUC was high, regardless of choroidal thickness; for eyes with CSC, the AUC decreased with increasing choroidal thickness (Fig. 2).

### Deep learning model

**Figure 3:**
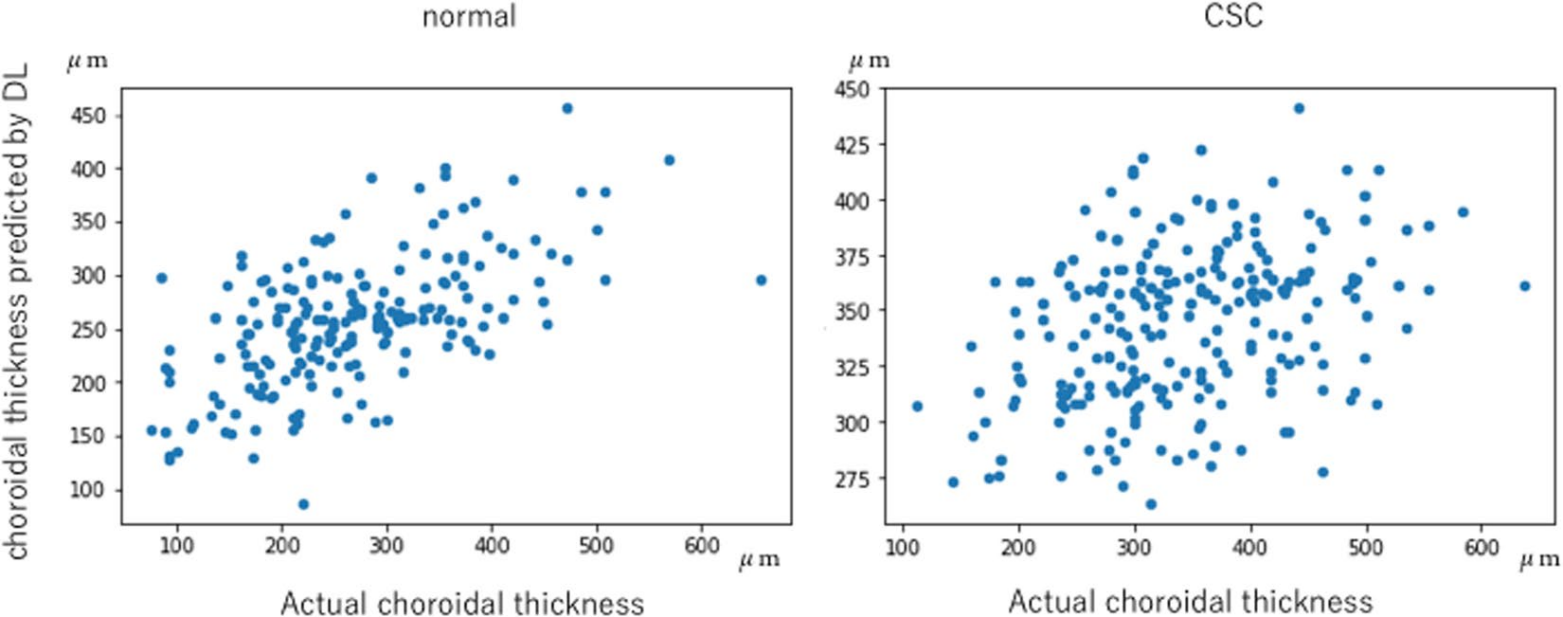
The horizontal axis represents the actual choroidal thickness, while the vertical axis represents the thickness as predicted by deep learning. The correlation coefficients (p values) were 0.68 (p < 0.001) and 0.48 (p < 0.001) for normal eyes (left) and eyes with central serous chorioretinopathy (CSC), respectively.

Deep learning enabled prediction of choroidal thickness, using colour fundus photographs. The correlation coefficient between the predicted and actual choroidal thickness values was 0.68 for normal eyes (p < 0.001); for eyes with CSC, the correlation coefficient was 0.48 (p < 0.001), which was lower than the coefficient for normal eyes (Fig. 3).

**Figure 4:**
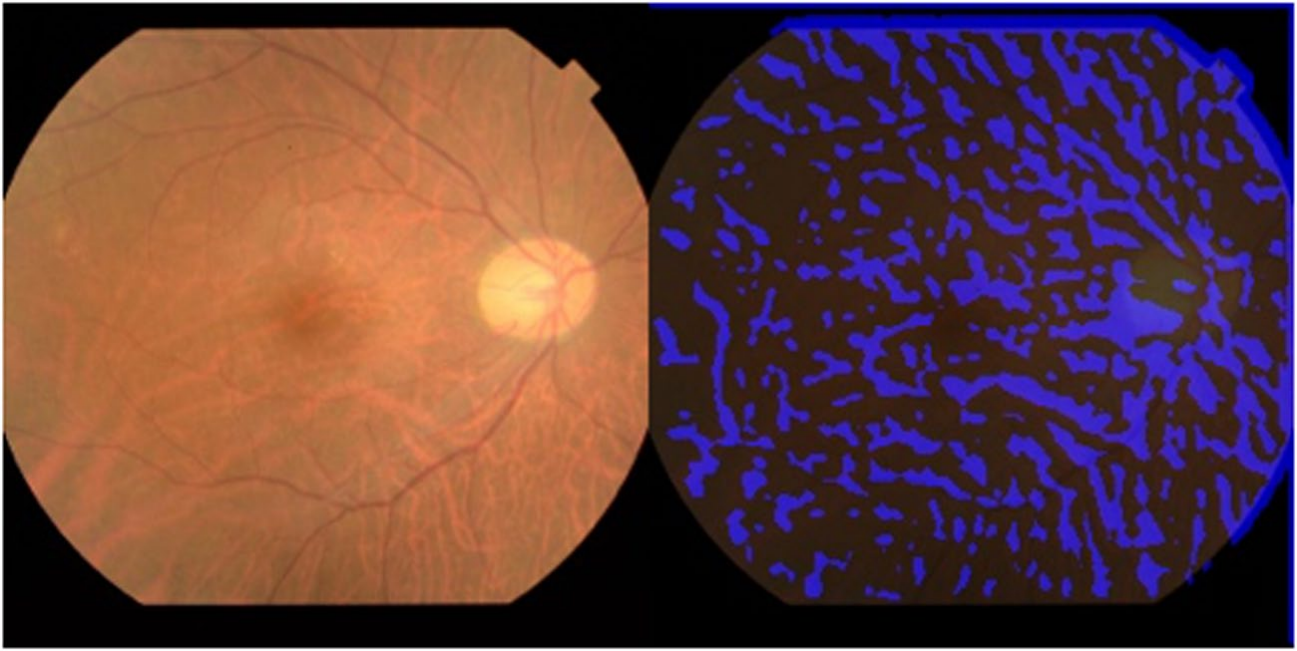
Points of interest on the heat map accumulated in choroidal vessels of the fundus.

Heat map images were created by overlaying heat maps of the focus site of the deep neural network. An example is presented in Fig. 4. Points of interest on the deep learning images were similar to the binarisation images, because some heat maps demonstrated accumulation in the choroidal vessels of the fundus.

## Discussion

In this study, we attempted to estimate choroidal thickness at the macula by using conventional fundus photographs. Advances in OCT have enabled observation of the choroid, but not all hospitals and clinics possess OCT devices. In contrast, colour fundus photography is very common and widely used in hospitals and clinics, as well as in health screening facilities. Because choroidal thickness has been associated with various macular diseases (e.g., pachychoroid-related disease and myopic chorioretinal atrophy), it may be useful to determine choroidal thickness automatically via fundus photographs, without an OCT device; the findings could be used to alert patients to the risks of macular disorder at the non-symptomatic stage of pachychoroid-related disease. The current study established the utility of conventional fundus photography for the estimation of choroidal thickness. Both the advanced deep learning method and image analysis were successful in estimating choroidal thickness.

In 1977, Delori *et al*. reported that monochromatic light at relatively long wavelengths could be used to observe choroidal vessels^13^. In the lightly pigmented fundus, choroidal vasculature was distinguishable under deep red (620–650 nm) light. In addition, retinal vessels were clearly observed at green wavelengths (540–580 nm). In the current digital era, separation of full-colour fundus photographs into red, green, and blue channels is a simple and convenient approach to obtain monochromatic renderings. As observed in the present study, a red-channel monochromatic image can render the choroidal vessels, if the retinal vessels are subtracted by using information from the green channel image. Recently, Kakiuchi *et al*. reported a similar attempt to depict choroidal vasculature using the 635 nm wavelength for ultra-widefield images, which yielded high reproducibility by indocyanine green angiography^14^. Finally, the CVAI obtained in our analysis showed an inverse correlation with choroidal thickness.

Deep learning techniques also enabled estimation of choroidal thickness from fundus photographs. Although the available data were relatively sparse, transfer learning (image augmentation) enabled efficient assessment of the characteristics of an image. Heat map images suggested that deep learning focused on the choroidal vascular image when estimating choroidal thickness. In the field of ophthalmology, deep learning systems have demonstrated accuracy in detection of diabetic retinopathy, glaucoma, and age-related macular degeneration from fundus photographs^15–17^. They also have demonstrated success in identification of disease features by OCT, including progression and treatment responses in chorioretinal diseases (e.g., age-related macular degeneration and diabetic macular oedema)^18^. Our results suggest that deep learning can be used to identify eyes with pachychoroid, directly from fundus photographs.

Notably, the estimation of choroidal thickness from fundus photographs was more difficult for eyes with CSC than for normal eyes. Both imaging analysis and deep learning showed lower accuracy in the estimation of choroidal thickness in eyes with CSC; in these eyes, visualisation of choroidal vasculature in the fundus became obscure. Indeed, the CVAI was lower for eyes with CSC than for normal eyes in this study. In contrast, Hirahara *et al*. reported that the choroidal vessel density obtained by binarising ultra-wide-field indocyanine green angiography images was higher for eyes with CSC^19^. The discrepancy between their findings and the present findings could be related to differences regarding direct and indirect detection of choroidal vessels on indocyanine green angiography images and fundus photographs.

Choroid is known to be thicker in eyes with CSC^1^. This is caused by an increase in choroidal vascular density, dilation of choroidal vasculature, and an increase in choroidal stroma^20^; Sonoda *et al*. observed an increase in choroidal stroma within the inner choroid on OCT analysis of eyes with CSC. Moreover, the choroidal stroma contains a large number of melanocytes with melanin^21^. Melanin distribution in the choroid might be altered in eyes with CSC, such that the inner choroid contains additional melanin because the outer choroid is filled with dilated choroidal vasculature. Thus, the increase in melanin content, which overlaps with choroidal vessels, might reduce the transparency of choroidal vessels in red wave-length colour photography. The presence of subretinal fluids might also affect the visibility of choroidal vessels.

Similar to CSC, Vogt–Koyanagi–Harada disease is known to involve thick choroid in the active stage, possibly due to infiltration by macrophages or other inflammatory cells in the choroidal stroma. Immediately before recurrence, fundus examinations reportedly show reduction of choroidal vessel density and OCT images show thicker choroid; in contrast, the appearance of choroidal vessels is more distinct in the fundus of myopic or aged eyes with thin choroid^9,22^. Therefore, the visibility of choroidal vessels in fundus photographs might be inversely correlated with choroidal thickness.

There were several limitations in this study. First, images with low contrast, brightness, and colour were eliminated due to difficulties in both binarisation and deep learning analyses. Second, red wavelength fundus photography can easily detect drusen as white and nevus as black, which greatly influences CVAI data; therefore, eyes with many drusen and nevus were excluded from analysis. Third, this study included a limited number of data sets, which might have affected the precision of the correlation. The accumulation of additional data would yield a more precise formula.

In conclusion, this study showed that fundus photos could be used to estimate choroidal thickness. Because colour fundus photography is a gold standard imaging tool in ophthalmology, our approach should aid in identification of patients with abnormal choroidal thickness before the development of ocular pathology.

## Methods

This study was conducted in compliance with the Declaration of Helsinki. The research protocols and implementation were approved by the Ethics Committee of Hyogo College of Medicine and Tsukazaki Hospital. Data were collected from patients who visited Department of Ophthalmology, Hyogo College of Medicine and Tsukazaki Hospital. Informed consent was obtained in the form of opt-out. All numerical data are expressed as the means ± standard errors of the mean. Comparisons were made using the Mann–Whitney U test.

The analysis was performed using colour fundus photographs (TRC-50DX, Topcon, Tokyo) with similar brightness, contrast, and colour balance characteristics, as well as choroidal thickness values from 100 normal eyes of 100 patients and 100 eyes of 100 patients with CSC who were examined in the Department of Ophthalmology, Hyogo College of Medicine and in Tsukazaki Hospital were used for the analysis. Diagnosis of CSC was made by fluorescein and indocyanine green angiography; all eyes with CSC exhibited subretinal fluid in the macula. Choroidal thickness under the fovea was measured by the enhanced depth imaging technique on an OCT device (Spectralis, Heidelberg, Germany)^4,23^.

### Image processing

**Figure 5:**
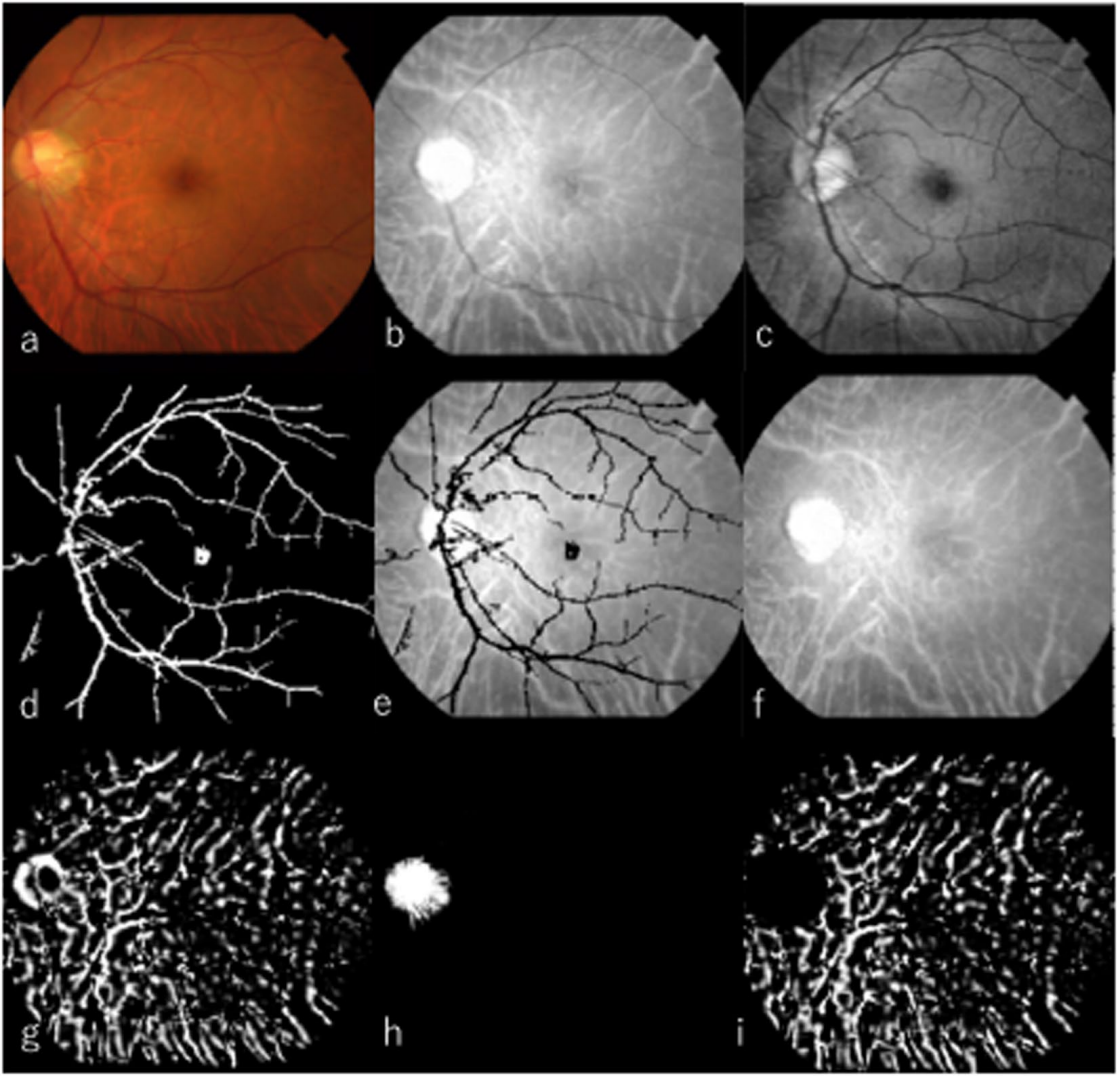
Retinal blood vessel elimination in an R-component image. (**a**) Original colour fundus photograph. (**b**) R-component of fundus image. (**c**) G-component after contrast limited adaptive histogram equalization (CLAHE) adaptation. (**d**) Binarisation of (**c**). (**e**) Following mask processing with substitution of (**d**) for (**b**), the blood vessels in the R-component image are hidden. (**f**) Blood vessel elimination image obtained by complementation of the blood vessel region. (**g**) Binarisation of (**f**). (**h**) Detection of optic disc. (**i**) Erasure of optic disc.

Each colour fundus photograph was separated into 8-bit RBG components. The R-component image was used in the detection of choroidal vessels; the G-component image was used in the subtraction of retinal blood vessels from the R-component image. First, the G-component image was binarised by the adaptive binarisation method with a weighted-average threshold using a Gaussian kernel superimposed on the R-component image; retinal blood vessels were subtracted in this step. The R-component image was binarised by using the same method, then compared with a G-component image from which retinal vessels had been subtracted; this produced the choroidal vessel-dominant image. To remove the strong optic disc signal, the logical product of the areas corresponding to the optic disc in binary images of all RGB components were merged and subtracted from the choroidal-vessel dominant image. Then the ratio of the number of white pixels to the total number of pixels in the image was calculated. This ratio was defined as CVAI (Fig. 5), using the following formula:

$${\rm{C}}{\rm{V}}{\rm{A}}{\rm{I}}={\rm{C}}{\rm{V}}{\rm{R}}/{\rm{I}}{\rm{R}}$$

where CVR is the number of pixels (i.e., area) of the choroidal blood vessel region, and IR is the number of pixels (i.e., area) of the imaging region.

### Deep learning

**Figure 6:**
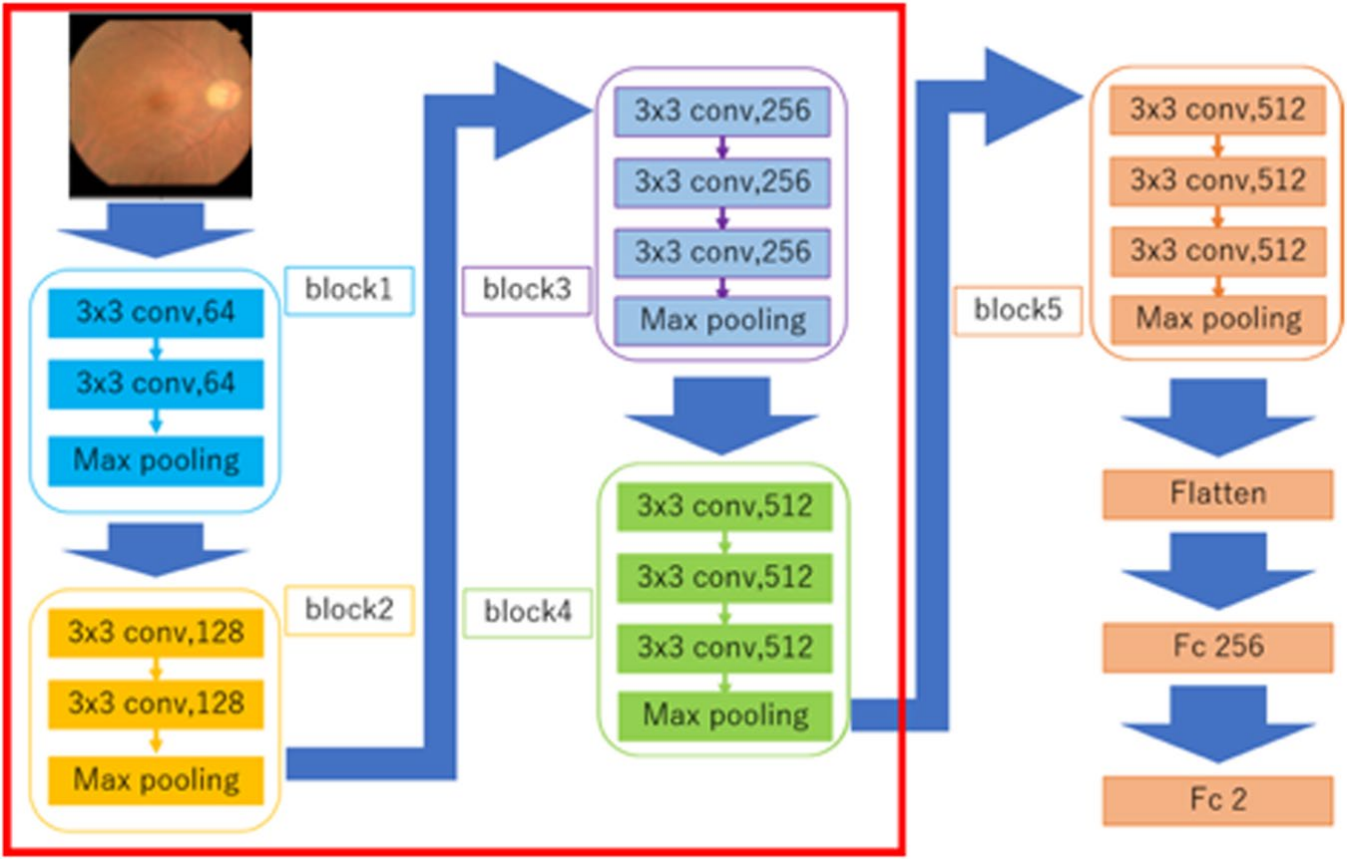
Overall architecture of the Visual Geometry Group-16 (VGG 16) model. VGG-16 comprises five blocks and three fully connected layers. Each block comprises some convolutional layers, followed by a max-pooling layer. After the output matrix has been flattened following block 5, there are two fully connected layers for binary classification. The deep neural network used ImageNet parameters as the default weights of blocks 1–4.

A deep convolutional neural network model was created and trained with the augmented training data with K-Fold Cross Validation (K = 5). Images of the training data were augmented by adjustment for brightness, gamma correction, histogram equalisation, noise addition, and inversion; thus, the amount of training data increased by six-fold. After training had been performed, the abilities of the models were analysed by using the validation data. Visual geometry group-16 was used as the convolutional neural network in the present study (Fig. 6)^24^.

The strides of the convolutional layers were 1 and the padding of the layers was ‘same’; therefore, the convolutional layers only captured the features of the image, and did not downsize the image. The activation function of the layers was ReLU, which enabled avoidance of the vanishing gradient problem^25^. The strides of the max pooling layers were 2; thus, the layers compressed the information of the image. After block 5, a flattened layer and two fully connected layers were used. The flattened layer removed spatial information from the extracted feature vectors, while the fully connected layers compressed the information from the previous layers. The activation function of the last fully connected layer was Linear. The performance evaluation items were the correlation coefficients of the values predicted by the neural network and the measured choroidal thickness.

### Heat map

Overlying heat map images of the deep neural network focus site were created by applying a gradient-weighted class activation mapping method to the corresponding fundus images^26^. The gradient-weighted class activation mapping method was used to maximise the outputs of the third convolutional layer pooling in block 3. The function in back-propagation steps for modification of loss of function was the rectified linear unit, which propagated only positive gradients. This process was performed using Python Keras-vis (https://raghakot.github.io/keras-vis/).

## Data Availability

The data are not available for public access because of patient privacy concerns, but are available from the corresponding author on reasonable request.
